# Football in Colleges

**Published:** 1894-01

**Authors:** 


BUFFALO MEDICAL AND SURGICAL JOURNAL

A MONTHLY REVIEW OF MEDICINE AND SURGERY.

*EDITORS:*

THOMAS LOTHROP, M. D. - - WM. WARREN POTTER, M. D.

All communications, whether of a literary or business character, should be addressed to the managing editor: 284 Franklin Street, Buffalo, N. Y.

Vol. XXXIV. JANUARY, 1894. No. 6.

### FOOTBALL IN COLLEGES.

The importance of physical exercise for the body is universally admitted; thinking men of all ages have recognized its value also as a factor in expanding and strengthening the intellect. A healthy mind in a healthy body is a proverbial phrase, but it is only within the last few decades that this somewhat worn dictum has been found to have a scientific base.

Late investigations have proved beyond cavil that not only does the mind depend upon the body, but that the most satisfactory condition of the body cannot coëxist with what is called a diseased mind. Whether there be or not, that which we call the soul, to sit in judgment on the actions or operations of the mind, it is certain, from investigations made and still making, that the latter not only acts upon the body, but is itself subject to the condition of the brain, governing and being governed by the movements and changes of its cells, and that health or disease depend on their normal or abnormal state.

There is an old story about a quarrel between the pendulum and the hands of a clock, and, if our memory is not at fault, the stomach and heart were once at loggerheads about the relative value of their tasks. Nothing was said, we believe, by the patient and silent nerves, that, after all, had to keep the whole in working order. These have at last found a voice, and their claim is not likely to be ignored in the future.

If mind and body are so inseparably connected, then it follows that proper physical exercise aims at the equal development and strengthening of all parts of the body. Various have been the means by which this object has been sought. There is difficulty in childhood, and but little in the early stages of adolesence. Running, skating, swimming, rowing, with all the movements of the body and diversions of the mind accompanying them, will, with proper diet, develop all that heredity or disease does not prevent; but there comes a time when the future citizen, on whose virtue and judgment his country depends, must seriously tone down to his mental education. Now, more than ever, he needs physical exercise and diversion to keep his brain and nervous system in good working order.

Thus far we anticipate no difference of opinion; it is in regard to what constitutes proper and rational amusement—for amusement must necessarily accompany exercise to benefit the mind —that we may diverge. Of course, it will be understood that what we have in mind relates to practices in the higher departments of education, where we expect to find sensible men and youths who would scorn to be called boys. Base ball and rowing have always been deemed legitimate modes of exercise and amusement, but of late there has been transported from England an abomination called football; not that it is particularly new even in our own country, though formerly it was not deemed the chief mark of distinction in our colleges. Now, owing to an emulation more honored in the breach than in the observance, some of these have acquired a notoriety scarcely less repugnant to the good sense of the community than that of professional athletes and bruisers.

In the contest thus inaugurated, mere physical strength and brutal weight must necessarily carry the day against higher and more refined organizations. If these contests could be confined to the coarser and less intellectual of the students, perhaps no great harm would accrue, but, through the *esprit de corps* which must necessarily exist in such institutions, more delicate youths, whose physique no more fits them for such rough usage than to match a Sullivan or a Mitchell, are drawn in sometimes to danger of life and limb. Nor is this a figure of speech, for besides the maimed and injured that with nearly every set-to require surgical aid, too often it happens that life itself is lost. Only a few days ago a youth, who might have become a blessing to his country, was overwhelmed by a brutal crowd and killed.

Briefly, there can be no excuse for such rough play among gentlemen, and when it comes to hiring professional athletes, the whole matter takes on a still more reprehensible face. The only apology offered by writers in newspapers for football contests in colleges amounts to this: they are not as bad as they are represented. A writer in one of our medical journals even comes to their rescue, yet he has a nice little catalogue of broken bones and other injuries to report, intending to minimize the dangers; but as he only refers to Harvard, where we ought to look for the most gentle students, since the Hub is so near, his list of injuries are not to be taken as an average. If it be true, as alleged, that football in colleges is practised only by crews selected for their prowess and special training, all argument for its continuance based on need of physical exercise in keeping up a healthy condition of mind and body falls to the ground.

This leads us to consider another side of the subject: Is there not danger that contests by proxies (*i. e.*, selections of a certain number of athletes,) may lead to neglect of regular and proper gymnastic exercises by those who most stand in need of them? To class football with boating and baseball is misleading; these may, it is true, be carried to dangerous excess, but they are, when properly carried on, exhibitions of skill and scientific training—not a vulgar rough and tumble pel mel. That boys should love such sport is easily understood, but it is difficult to see how it can be popular with men of cultivated manners. It may be that our protest will avail nothing against a practice so popular in our collegiate institutions, that advance in science or learning is no longer the test of their products, but rather the physical endurance or athletic powers of their respective *teams.* Whole sides of our most important newspapers are filled with the details of these performances, and their young lady friends are not ashamed to witness these exhibitions and applaud to the echo. Still, it were criminal to withhold our censure when we know that such games are fraught with physical danger, and believe them to be contrary to the best ethical culture.

